# Investigation of FOXM1 as a Potential New Target for Melanoma

**DOI:** 10.1371/journal.pone.0144241

**Published:** 2015-12-07

**Authors:** Azusa Miyashita, Satoshi Fukushima, Satoshi Nakahara, Junji Yamashita, Aki Tokuzumi, Jun Aoi, Asako Ichihara, Hisashi Kanemaru, Masatoshi Jinnin, Hironobu Ihn

**Affiliations:** Department of Dermatology and Plastic Surgery, Faculty of Life Sciences, Kumamoto University, 1-1-1 Honjo, Chuo-ku, Kumamoto, Japan; Rutgers University, UNITED STATES

## Abstract

Recent studies have shown that immunotherapies and molecular targeted therapies are effective for advanced melanoma. Non-antigen-specific immunotherapies such as immunocheckpoint blockades have been shown to be effective in the treatment of advanced melanoma. However, the response rates remain low. To improve their efficacy, they should be combined with antigen-specific immunotherapy. Elevated expression of the transcription factor, Forkhead box M1 (FOXM1), has been reported in various human cancers, and it has been shown to have potential as a target for immunotherapy. The purpose of this study was to investigate the FOXM1 expression in human melanoma samples and cell lines, to evaluate the relationship between the FOXM1 expression and the clinical features of melanoma patients and to investigate the association between the FOXM1 and MAPK and PI3K/AKT pathways in melanoma cell lines. We conducted the quantitative reverse transcription PCR (qRT-PCR) and Western blotting analyses of melanoma cell lines, and investigated melanoma and nevus tissue samples by qRT-PCR and immunohistochemistry. We performed MEK siRNA and PI3K/AKT inhibitor studies and FOXM1 siRNA studies in melanoma cell lines. We found that FOXM1 was expressed in all of the melanoma cell lines, and was expressed in 49% of primary melanomas, 67% of metastatic melanomas and 10% of nevi by performing immunohistochemical staining. Metastatic melanoma samples exhibited significantly higher mRNA levels of FOXM1 (*p* = 0.004). Primary melanomas thicker than 2 mm were also more likely to express FOXM1. Patients whose primary melanoma expressed FOXM1 had a significantly poorer overall survival compared to patients without FOXM1 expression (p = 0.024). Downregulation of FOXM1 by siRNA significantly inhibited the proliferation of melanoma cells, and blockade of the MAPK and PI3K/AKT pathways decreased the FOXM1 expression in melanoma cell lines. In conclusion, FOXM1 is considered to be a new therapeutic target for melanoma.

## Introduction

Malignant melanoma is one of the most aggressive skin cancers, and its incidence has been gradually increasing [1]. Malignant melanoma is responsible for most skin cancer-related deaths. Recent studies have shown that immunotherapies and molecular targeted therapies are effective for advanced melanoma. Ipilimumab (a fully human monoclonal antibody against cytotoxic T-lymphocyte antigen 4) has demonstrated consistent activity against advanced melanoma [2]. The survival curve began to plateau around the third year of treatment; the three-year survival rate was 20% [3]. Nivolumab (anti–programmed death 1 antibodies) was associated with objective responses in 30–40% of patients with metastatic melanoma [4]. The combination of nivolumab and ipilimumab resulted in an objective response that ranged from 50 to 60% [4]. On the other hand, vemurafenib (a BRAFV600E kinase inhibitor), has remarkable antitumor activity in patients with BRAFV600E-mutated melanoma. The median progression-free survival that was observed with the combination of BRAF and MEK inhibition is similar to that recently reported with combined nivolumab and ipilimumab (11.7 months in patients with a *BRAF* mutation) [4]. However, the effects of the inhibitor therapies are limited by the onset of drug resistance, which occurs within a period several months. Thus, it can be said that the therapies for advanced melanoma have improved greatly. However, there is some area for improvement in both types of therapy. We are of the opinion that antigen-specific immunotherapy should be used together with immunocheckpoint blockades. We have focused our attention on Forkhead box M1 (FOXM1) as a target for anti-cancer immunotherapy in melanoma.

FOXM1 is a member of a family of transcription factors that regulate the expression of genes essential for cell proliferation and transformation and are implicated in tumorigenesis and tumor progression. FOXM1 is a key cell cycle regulator of both the transition from the G1 phase to the S phase and the progression to mitosis [5, 6]. FOXM1 accumulates mainly in the cytoplasm at the late G1 and S phases, and nuclear translocation of the protein occurs before entry of the cells into the G2-M phase following cyclin E-CDK2 and Raf-MEK-ERK-mediated phosphorylation [7]. Furthermore, it has been shown that the loss of FOXM1 expression in cancer cell lines results in mitotic spindle defects, delays in mitosis and the induction of mitotic catastrophe [6]. Thus, FOXM1 is essential for cancer cell growth and survival. Additionally, tumor cells overexpressing FOXM1 are resistant to apoptosis and the premature senescence induced by oxidative stress, which has strong implications for resistance to chemotherapy [8].

The abnormal upregulation of FOXM1 is involved in the oncogenesis of various human cancers, including breast, lung, bile duct, prostate, brain and pancreatic cancers, in addition to basal cell carcinoma (BCC) and head and neck squamous cell carcinoma (SCC) [9–15]. Yokomine *et al*. reported that FOXM1 is overexpressed in various cancers based on a cDNA microarray analysis, and they revealed that FOXM1-derived peptides binding to HLA-A2 had the capacity to induce CTLs [9]. The authors also analyzed normal tissues, and showed that FOXM1 is expressed only in the testes, thymus, small intestine and colon in normal adult humans [9]. The ideal targets for anti-cancer immunotherapy should have two main characteristics: 1) the antigens should be overexpressed in cancer tissues, but not in normal tissues because high expression in normal tissues will result in the autoimmune responses; 2) the antigens should have an essential function in cancer cell growth and survival. In immunotherapy, antigen loss is an important problem that remains to be solved. Even if the cancer cells lose those kinds of the antigen to escape from the immune system, then cell growth and survival will be inhibited. FOXM1 is therefore considered to be a suitable target for anti-cancer immunotherapy. A multiple peptide cocktail vaccine (KOC1, FOXM1 and KIF20A) has already been tested in patients with refractory pediatric sarcoma in a phase I study. We hypothesize that the combination of immunocheckpoint blockades and antigen specific immunotherapy, utilizing FOXM1, will make immunocheckpoint blockades more effective. Additionally, a FOXM1 inhibitor, a thiazole antibiotic, siomycin A, was reported to induce apoptosis in metastatic melanoma cell lines that correlated with the downregulation of FoxM1 [16]. Therefore, FOXM1 could be a target for not only immunotherapy, but also molecular targeted therapy. Several studies have previously reported that FOXM1 is overexpressed in human melanoma cell lines [16–18], however, to the best of our knowledge there have been no previous studies in human melanoma tissue samples. In this study, we aimed to determine expression of FOXM1 in human melanoma samples and to evaluate the relationship between the FOXM1 expression and the clinical features of melanoma patients.

The MAPK and PI3K/AKT pathways represent the most frequently mutated signaling pathways in human cancers, including malignant melanoma. It has been reported that up to 70% of melanomas carry the BRAFV600E mutation [19], and 70% have elevated AKT phosphorylation [20]. The high prevalence of dysregulation of these two pathways provides a rationale for the development of target-based therapeutics for treatment. FOXM1 has been shown to have cross-talk with the MAPK pathway in malignant melanoma [17, 18]. The cross-talk of the AKT pathway with the FOXM1 pathway has been demonstrated [21, 22]. AKT can control FOXM1 expression in osteosarcoma [21], and the downregulation of AKT by siRNA has been shown to inhibit FOXM1 expression, whereas the overexpression of AKT has been shown to increase FOXM1 expression in prostate cancer [15]; however, its role in melanoma cells has not been reported. To our knowledge, this is the first report that has investigated the association between the FOXM1 and PI3K/AKT pathways in melanoma cells.

## Materials and Methods

### Clinical assessment and patient characteristics

Tissue samples of melanomas and nevi were obtained during routine diagnostic procedures. A total of 20 benign nevi were obtained from 20 patients (10 males and 10 females), whose ages ranged from one to 86 years (mean: 44 years). Histologically, the samples included junctional, compound and intradermal variants. A total of 43 primary cutaneous melanomas were obtained from 43 patients (21 males and 22 females), whose ages ranged from 36 to 93 years (mean: 68 years). The primary cutaneous melanomas had Clark’s levels ranging from I to V, and Breslow depths ranging from *in situ* to 53 mm. A total of 13 metastatic melanomas were obtained from nine patients (three males and six females), whose ages ranged from 52 to 84 years (mean: 71 years). Four metastatic melanomas were localized to the regional lymph nodes, while the others were obtained from skin metastases. We gathered patient information from the medical records to determine the clinical stage according to the American Joint Committee on Cancer (AJCC) Cancer Staging Manual, 7th edition staging system for melanoma of the skin [23].

Primary melanomas are classified into four clinical and pathological subtypes: lentigo maligna melanoma (LMM), superficial spreading melanoma (SSM), nodular melanoma (NM) and acral lentiginous melanoma (ALM). Institutional review board (Faculty of Life Sciences Kumamoto University clinical research and medical technology ethics board) approved this study. The written informed consent was obtained from the patients before they were enrolled in this study. And also the written informed consent was obtained from the guardians on behalf of the minors/children enrolled in our study. The study was performed in accordance with the Declaration of Helsinki.

### Cell lines and culture conditions

Human melanoma cell lines were maintained in DMEM medium supplemented with 20% fetal bovine serum (FBS) in a 5% CO_2_ atmosphere at 37°C. The pancreatic cancer cell line, PANC1, was maintained in DMEM medium supplemented with 10% FBS in a 5% CO_2_ atmosphere at 37°C. Primary normal human epidermal melanocytes (NHEM) in CSF-4HM-500D culture medium supplemented with human melanocyte growth supplements were maintained in a 5% CO_2_ atmosphere at 37°C. The human melanoma cell lines were kindly provided by the Cell Resource Center for Biomedical Research Institute of Development, Aging and Cancer, Tohoku University (Sendai, Japan), Dr. Y. Kawakami (Keio University; Tokyo, Japan) and the ATCC (Manassas, VA, USA). The NHEM were purchased from Promo Cell (Heidelberg, Germany) and the ATCC (Manassas, VA, USA).

### Reverse transcription-PCR

Total RNA was extracted from the tissues and cell lines using the RNeasy kit (Macherey-Nagel, Düren, Germany) according to the manufacturer’s instructions, and purified RNA was then reverse transcribed into cDNA using the PrimeScript RT reagent Kit^®^ (TaKaRa, Shiga, Japan), as described in the manufacturer's protocol. Equal aliquots of cDNA were used for quantitative RT-PCR (qRT-PCR) employing the SYBR^®^ Premix Ex Taq ™ II (TaKaRa, Shiga, Japan), according to the manufacturer’s protocol. The qRT-PCR primers used for FOXM1 and MEK1 were purchased from Takara (Shiga, Japan). The GAPDH primers were purchased from QIAGEN (Tokyo, Japan). The cDNA samples obtained from the cell lines were used as templates for semi-qRT-PCR under the following cycling conditions: 40 cycles of denaturation for five seconds at 95°C, annealing for 10 seconds at 58°C and extension for 20 seconds at 72°C. The PCR products were separated via electrophoresis on 2% agarose gels, stained with ethidium bromide and visualized with the Gel Documentation System. The semi-qRT-PCR primer sequences used in the study were: 5’-CACCCCAGTGCCAACCGCTACTTG-3’ and 5’-AAAGAGGAGCTAT-CCCCTCCTCAG-3’, which can detect three splicing variants: FOXM1a, FOXM1b and FOXM1c [9].

### MicroRNA extraction and quantitative real-time polymerase chain reaction

Total RNA was extracted from cell lines using the RNeasy kit (Macherey-Nagel, Düren, Germany) according to the manufacturer’s instructions. The cDNA was synthesized from total RNA using a Mir-X miRNA First Strand Synthesis kit (Takara Bio Inc.). For quantitative PCR, the primers for miR-370 were designed on the basis of the information provided in the miRBase (http://www.mirbase.org): GCCTGCTGGGGTGGAACCTGGT. Primers and templates were mixed with SYBR Advantage qPCR Premix (Takara Bio Inc.), and cDNAs were amplified for 40 cycles of denaturation for 5 s at 95°C and annealing for 20 s at 60°C.

### Western blotting analysis

A Western blotting analysis was performed as described previously [24]. The primary antibodies were: polyclonal rabbit anti-FOXM1 antibody (Santa Cruz Biotechnology, CA, USA, 1:100), monoclonal rabbit anti-phospho-AKT (ser473) antibody (Cell Signaling Technologies, Tokyo, Japan, 1:500), monoclonal rabbit anti-AKT (pan) antibody (Cell Signaling Technologies, Tokyo, Japan, 1:500), monoclonal rabbit anti-phospho-MEK antibody (Cell Signaling Technologies, Tokyo, Japan, 1:1000) monoclonal rabbit anti-MEK antibody (Cell Signaling Technologies, Tokyo, Japan, 1:1000). β-actin was used as a loading control.

### Immunohistochemical analysis

Immunohistochemical analyses were performed as described previously [9]. Sections of paraffin-embedded melanomas and nevus tissue samples were stained with a monoclonal mouse anti-FOXM1 antibody (clone 3A9; Abnova, Taipei, Taiwan), monoclonal mouse anti-BRAFV600E antibody (clone VE1; Spring Bioscience, Pleasanton, CA) and monoclonal rabbit anti-phospho-AKT (Ser473) antibody (Cell Signaling Technologies, Tokyo, Japan). An isotype monoclonal mouse antibody (clone MG2a-53; abcam, Tokyo, Japan) and an isotype monoclonal rabbit antibody (Cell Signaling Technologies, Tokyo, Japan) were used as negative controls. The slides were mounted using aqueous medium and viewed under a microscope. The intensity of staining was classified as (-) (the same or weaker than the adjacent epidermis) or (+) (stronger than the adjacent epidermis). The samples were divided into two groups (positive or negative for FOXM1) according to the results of immunostaining, and the positive rate of FOXM1 was determined. Stained sections were scored according to the percentage of stained melanoma cells: 75–100%, 50–74%, 25–49%, 1–24% or negative. The samples were evaluated independently by two observers (A.M. and S.F.) in a blinded manner.

### Gene silencing using small interfering RNA (siRNA)

FOXM1-specific siRNA was purchased from SIGMA-ALDRICH (MO, USA), MEK1-specific siRNA was purchased from Cell Signaling Technologies (Tokyo, Japan) and scrambled control siRNA was purchased from Thermo Scientific Dharmacon (Kanagawa, Japan). Human malignant melanoma cell lines were transfected using the Lipofectamine RNAiMAX transfection reagent (Invitrogen Corporation, Carlsbad, CA).

### Cell proliferation assays

We performed the BrdU cell proliferation assay to confirm whether the downregulation of FOXM1 by transfection of FOXM1 siRNA could inhibit melanoma cell proliferation. CycLex Cellular BrdU ELISA Kit was purchased from CycLex. (Nagano, Japan). Melanoma cell lines were transfected with FOXM1-specific siRNA and scrambled control siRNA using the Lipofectamine RNAiMAX transfection reagent. After a 72-h incubation, we performed the BrdU cell proliferation assay by means of CycLex Cellular BrdU ELISA Kit. The experiments were conducted four times.

### MAPK and PI3K/AKT signaling pathway blockade in melanoma cells

To block the MAPK signaling pathway, melanoma cell lines were transfected with MEK1 siRNA (Cell Signaling Technology, Tokyo, Japan), using the Lipofectamine RNAiMAX transfection reagent. The scrambled control siRNA served as a control. To block the AKT signaling pathway, the PI3K inhibitor, LY294002 (Calbiochem, La Jolla, CA), and an AKT inhibitor (Calbiochem, La Jolla, CA) were added directly to the culture medium of the melanoma cells. DMSO served as a control.

### Statistical analysis

The statistical analyses were carried out using Mann-Whitney's U-test, the Kruskal-Wallis test, a 2x2 contingency table and the log-rank test. A *p*-value < 0.05 was considered to be statistically significant.

## Results

### Expression levels of FOXM1 in the melanoma cell lines

**Fig 1:**
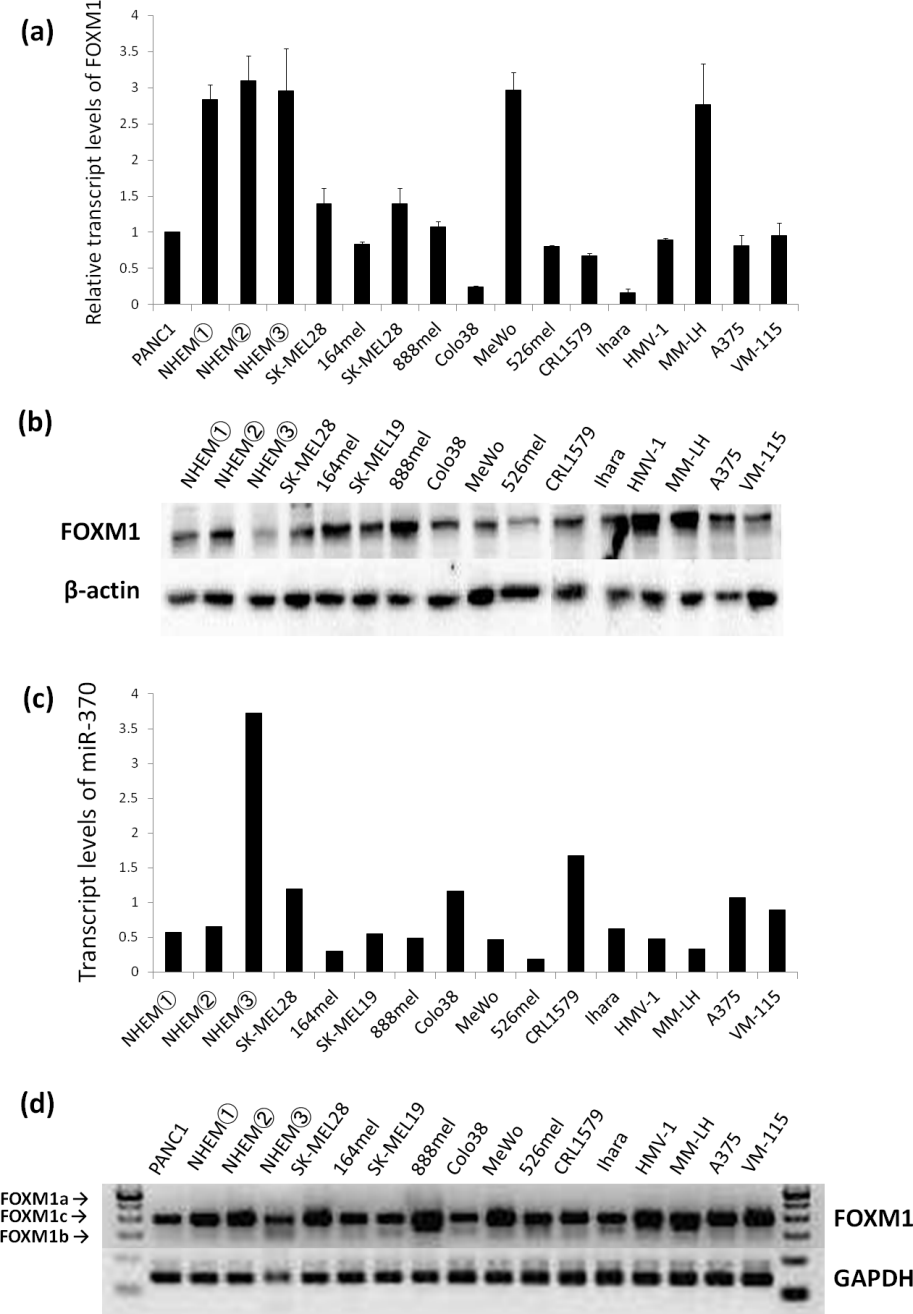
The expression of FOXM1 mRNA and protein in malignant melanoma cell lines, NHEM cells and PANC1 cells. PANC1 cells were used as a positive control. (a) The results of the quantitative RT-PCR analyses of FOXM1 mRNA expression. The relative transcript levels of FOXM1 normalized to the level in PANC1 are shown. (b) The expression of FOXM1 proteins in the malignant melanoma cell lines and NHEM. (c) The results of the quantitative RT-PCR analyses of the miR-370 mRNA expression. (d) The results of the semiquantitative RT-PCR using primers that can detect three splicing variants: FOXM1a (472bp), FOXM1b (323bp) and FOXM1c (368bp).

We first performed a qRT-PCR analysis of the FOXM1 expression in 13 malignant melanoma cell lines, NHEMs and a human pancreatic cancer cell line, PANC1, as a positive control. PANC1 cells were previously reported to have a high expression of FOXM1 [13]. As shown in Fig 1A, all of the malignant melanoma cell lines and NHEMs exhibited comparable expression levels of FOXM1 to the PANC1 cells. Furthermore, we added a semi-qRT-PCR analysis of FOXM1 to examine the expression of FOXM1 isoforms in the melanoma cell lines and NHEMs. The FOXM1 gene contains 10 exons, two of which (Va and VIIa) are alternatively expressed, giving a rise to three differentially expressed mRNA isoforms: FOXM1a, FOXM1b and FOXM1c [9, 14]. FOXM1a contains both alternative exons, FOXM1b contains none of the alternative exons and FOXM1c contains only exon Va [25]. Due to the absence of VIIa, which is the inhibitory sequence, FOXM1b and FOXM1c exhibit transactivating activity. The presence of VIIa in FOXM1a makes FOXM1a transcriptionally inactive [25]. It has been reported that the expression of the FOXM1b isoform is increased in BCCs and SCCs [14]. As shown in Fig 1D, we found that FOXM1c is the primary FOXM1 isoform in human melanoma cell lines and NHEMs.

We also performed a Western blot analysis of the melanoma cell lines and NHEMs, and found that the expression levels of FOXM varied from one cell line to another. All of the NHEMs showed a high level of FOXM1 mRNA (Fig 1A), however the protein levels of FOXM1 were downregulated in one NHEM (NHEM③) (Fig 1B). It can be said that some melanocytes express high protein levels of FOXM1 and some express low levels *in vitro*. Additionally, we investigated the expression levels of microRNAs in NHEMs and melanoma cell lines. MicroRNAs are a family of small noncoding RNAs that are important negative regulators of posttranscriptional gene expression, which eventually promote the degradation or translation suppression of target mRNAs. It has been reported that FOXM1 is a direct target of hsa-miR-370, and it was shown that the mRNA level of miR-370 was decreased in gastric cancer samples compared to normal tissue [26]. We observed that the expression of miR-370 in NHEM③, which expressed low protein levels of FOXM1, was higher than that in the other NHEMs and in all of the melanoma cell lines (Fig 1C). Therefore, one possible mechanism underlying the discrepancy between the mRNA and protein levels of FOXM1 in NHEM3 could be due do the high level of miR-370 in these cells. For the other NHEM and melanoma cell lines, we cannot talk about discrepancy.

### Detection of FOXM1 mRNA in the tissue samples

**Fig 2:**
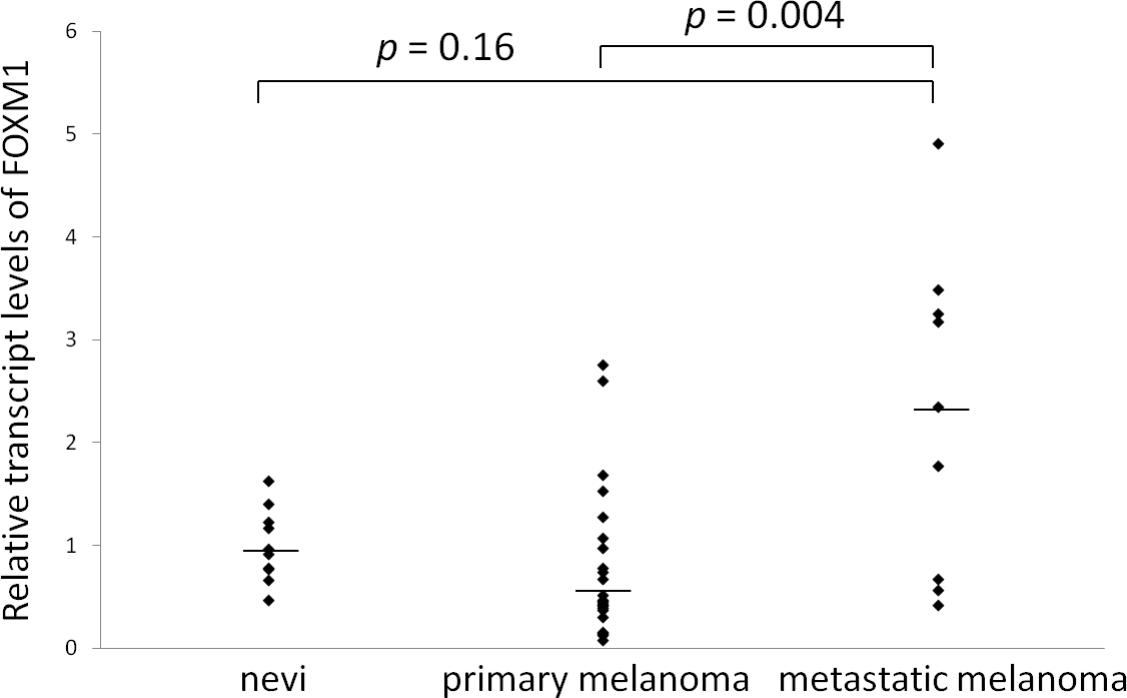
The results of the quantitative RT-PCR analyses of melanoma and nevus tissue samples. The FOXM1 expression (normalized to GAPDH) in the patients with primary melanoma (n = 25), metastatic melanoma (n = 9) and nevi (n = 10) is shown. The bars indicate the median values.

A quantitative RT-PCR analysis was performed in the primary melanoma, metastatic melanoma and nevus samples. As shown in Fig 2, the metastatic melanoma samples exhibited significantly higher expression levels of FOXM1 compared to the primary melanoma samples when the expression was normalized to GAPDH (*p* = 0.004). There was no statistically significant difference between nevi and metastatic melanoma (*p* = 0.14).

### Immunohistochemical analysis of FOXM1 in tissue samples

**Fig 3:**
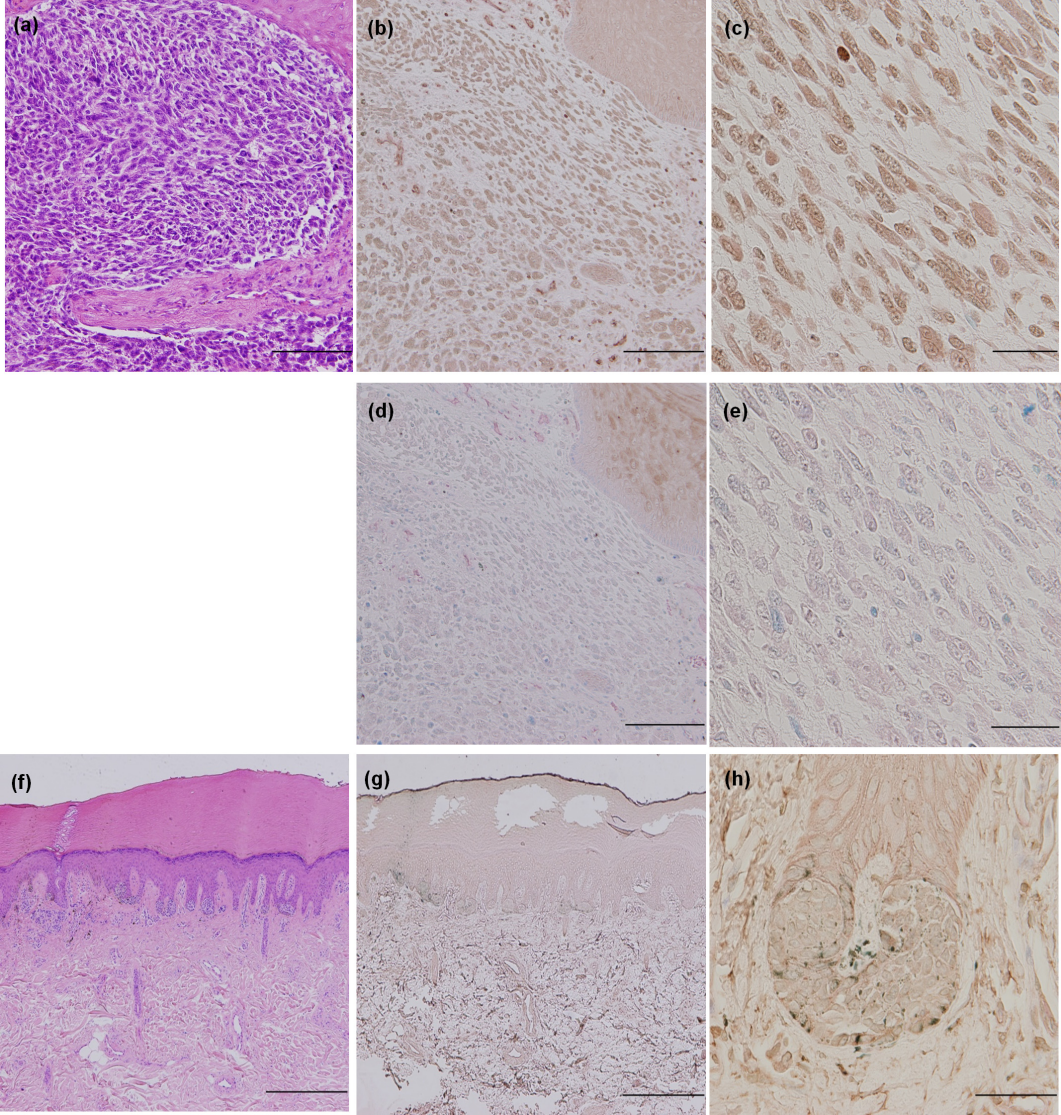
Immunohistochemical staining for FOXM1. Representative images of the immunohistochemical staining for FOXM1 in primary malignant melanoma (a, b, c, d, e) and nevus tissues samples (f, g, h). Hematoxylin and eosin staining (a, f: × 40) and FOXM1 immunohistochemistry (b, g: × 40, c, h: × 400). Negative controls using an isotype monoclonal antibody were presented in d and e (d: × 40, e: × 400). Melanin granules are indicated by blue staining, although they did not exhibit FOXM1 expression. Bars: 500 μm (a, b, d, f, g), 50 μm (c, e, h).

An immunohistochemical analysis of the FOXM1 expression in 43 primary cutaneous melanoma, 12 metastatic melanoma and 20 melanocytic nevus tissue specimens was performed. Representative examples are shown in Fig 3A–3H, and negative controls using an isotype monoclonal antibody are presented in Fig 3D and 3E. The melanoma cells positive for FOXM1 displayed homogenous cytoplasmic staining. The results of the immunohistochemical analysis are summarized in Table 1. The samples were divided into two groups (positive or negative for FOXM1) according to the immunostaining results, and the positive rates of FOXM1 detection were described. FOXM1 was highly expressed in the melanoma samples; however, low-level expression was observed in the melanocytic nevus samples. Twenty-one of the 43 primary melanomas (49%) and eight of the 12 metastatic melanomas (67%) were positive for FOXM1, 55% of the positive cases showed 75–100% staining of the melanoma cells, followed by 21% of cases showing 1–24%, 17% of cases showing 25–49% and 7% of cases showing 50–74%. Two of the 20 melanocytic nevi (10%) were positive for FOXM1. The melanoma samples therefore exhibited significantly higher positivity for FOXM1 than the melanocytic nevus samples (*p* = 0.0009). There were no correlations between positivity for FOXM1 and the histological type of melanoma or the AJCC stage.

**Table 1:** The results of the immunohistochemical analysis of FOXM1.

	n	Positive	Negative	Positive rate (%)
Nevi	20	2	18	10
Primary melanomas	43	21	22	49
Metastatic melanomas	12	8	4	67
LMM	8	3	5	37
SSM	9	5	4	55
NM	10	6	4	60
ALM	16	7	9	44
Stage 0	6	3	3	50
Stage I	8	3	5	37
Stage II	14	6	8	43
Stage III	12	8	4	67
Stage IV	3	1	2	33

LMM, lentigo maligna melanoma; SSM, superficial spreading melanoma; NM, nodular melanoma; ALM, acral lentiginous melanoma; n, number

Additionally, we performed an immunohistochemical analysis of BRAFV600E and phosphorylated-AKT in sections of paraffin-embedded melanomas and nevus tissue samples. Previous studies have shown that FOXM1 is modulated by the Raf/MEK/MAPK pathway and that it may mediate the G2/M regulatory effect of Raf/MEK/MAPK signaling [24]. The cross-talk of FOXM1 with the PI3K/AKT pathway has also been demonstrated [21, 22]. AKT can control FOXM1 expression in osteosarcoma [21], and the downregulation of AKT by siRNA has been shown to inhibit FOXM1 expression; whereas the overexpression of AKT increased FOXM1 expression in prostate cancer [15]. We therefore conducted an immunohistochemical analysis to confirm the correlation between the expression levels of FOXM1 and the BRAF mutation status and/or the pAKT status in melanoma tissue specimens. As shown in Table 2, we found that 55.2% of cases positive for FOXM1 were also positive for BRAFV600E, and 27.6% of the cases positive for FOXM1 were also positive for phospho-AKT. These results showed that the expression levels of FOXM1 correlate with the BRAF mutation status and/or the status of AKT phosphorylation in patient samples. We think our results fit with the concept of previous reports.

**Table 2:** The results of the immunohistochemical analysis of FOXM1, BRAFV600E and p-AKT.

	Mutated BRAF (n = 20)	Upregulated p-AKT (n = 12)
FOXM1-positive (n = 29)	55.2% (n = 16)	27.6% (n = 8)
FOXM1-negative (n = 27)	14.8% (n = 4)	14.8% (n = 4)

### The correlation between the FOXM1 expression and tumor thickness

The primary malignant melanomas were divided into two groups based on whether they were less than and greater than 2.00 mm in tumor thickness. Patients with stage 0 disease were excluded from this study. There was a significant difference in the FOXM1 positivity between the two groups. The number of patients with ≧ 2.01 mm thickness was significantly higher than that with ≦ 2.00 mm thickness among the FOXM1-positive cases (*p* = 0.046) (Table 3).

**Table 3:** The correlation between FOXM1 expression and the tumor thickness.

	≦ 2.00 mm thickness	≧ 2.01 mm thickness	*p* value
FOXM1 positive (n = 18)	3	15	0.046
FOXM1 negative (n = 19)	9	10	

*p* < 0.05 was considered statistical significance

### The correlation between the FOXM1 expression and overall survival

**Fig 4:**
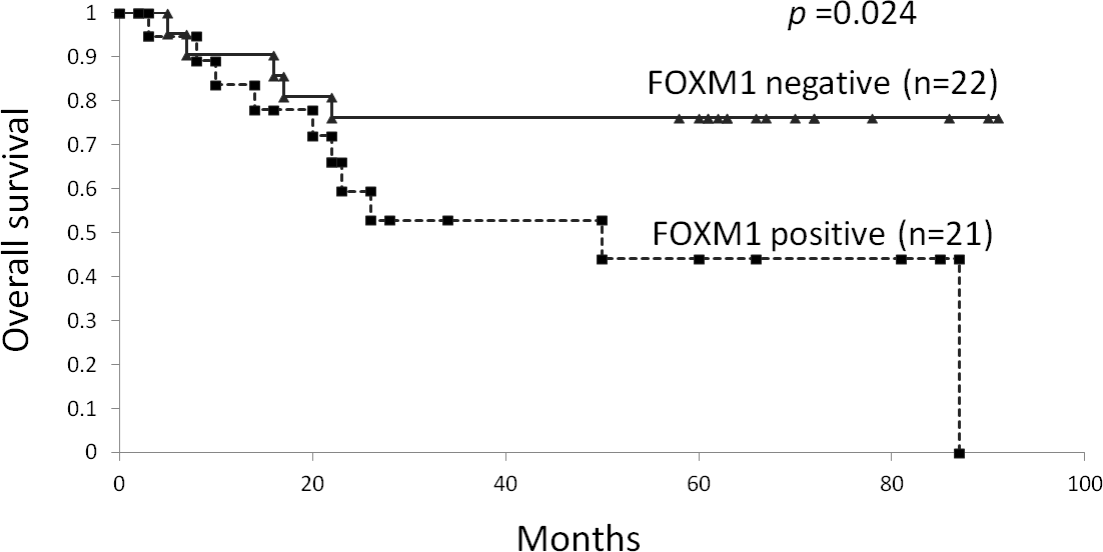
The relationship between FOXM1 expression and the overall survival. A comparison of the overall survival between the patients positive for FOXM1 and those negative for expression, as determined using immunohistochemical staining. The *p*-values were determined using the log-rank test.

Fig 4 shows the overall survival rate for the malignant melanoma patients estimated using the Kaplan-Meier method. We divided the patients into two groups based on the FOXM1 expression in the primary melanoma, as determined by immunohistochemical staining. The patients whose primary melanoma expressed FOXM1 had a significantly poorer overall survival compared to patients without FOXM1 expression (p = 0.024).

### The downregulation of the FOXM1 expression by siRNA inhibits cell growth

**Fig 5:**
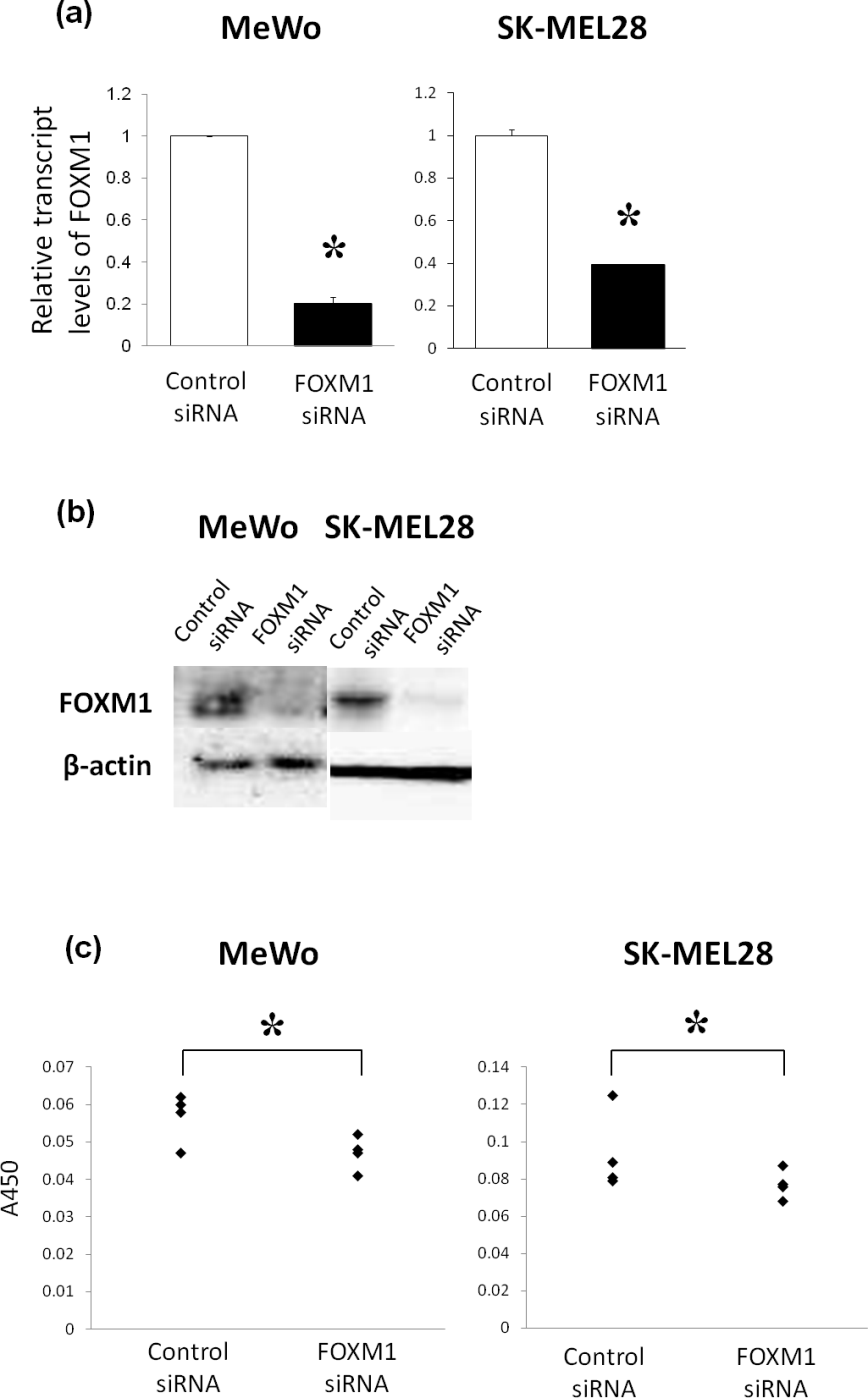
Downregulation of the FoxM1 expression by siRNA inhibited melanoma cell growth. The human melanoma cell lines, MeWo and SK-MEL28, were transfected with control and FOXM1 siRNA. Twenty-four hours after treatment, the quantitative RT-PCR analyses were carried out (a,). Seventy-two hours after treatment, a Western blotting analysis and the BrdU cell proliferation assay were performed (b, c). The *p*-values were determined using the Mann–Whitney U-test. * *p* < 0.05.

To determine whether FOXM1 could be an effective therapeutic target for malignant melanoma, the effects of FOXM1-specific siRNA on the proliferation of human malignant melanoma cell lines, MeWo and SK-MEL28, were examined. We performed a quantitative RT-PCR analysis and a Western blotting analysis to confirm the efficacy of FOXM1-specific siRNA. We observed that both FOXM1 mRNA and protein levels were decreased when FOXM1-specific siRNA was transfected into the melanoma cells (Fig 5A and 5B). And we found that the downregulation of FOXM1 expression significantly inhibited melanoma cell proliferation (Fig 5C).

### Blocking the MAPK pathway downregulates FOXM1 expression

**Fig 6:**
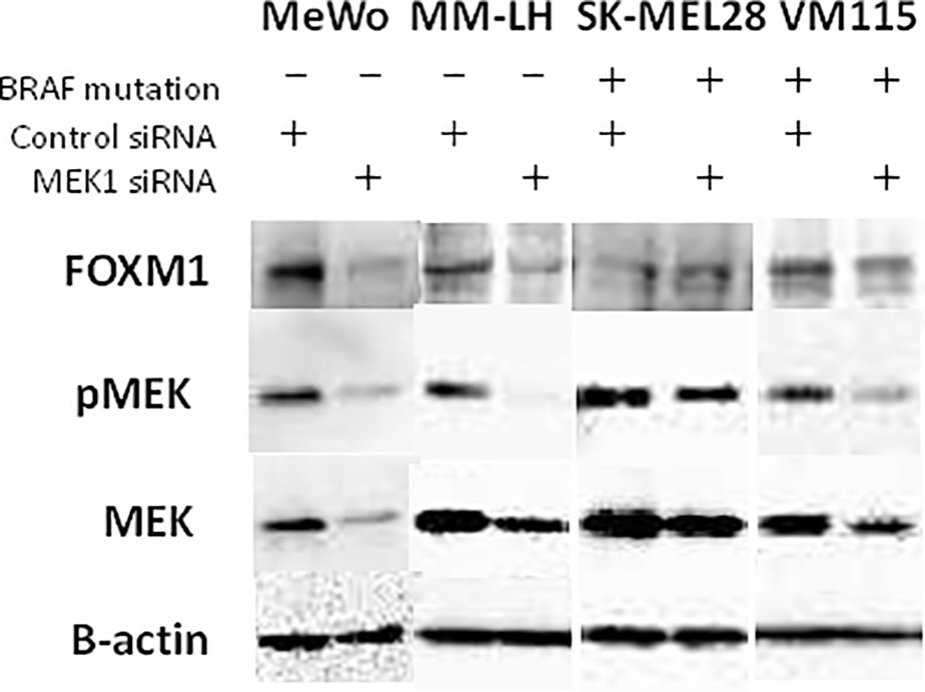
MAPK signaling pathway blockade in melanoma cells using a MEK1 siRNA. The expression of FOXM1 was assessed using a Western blotting analysis. The human melanoma cell lines were treated with 10 μM of MEK1 siRNA for 72 hours.

Next, we conducted an MEK siRNA analysis to determine whether FOXM1 could be a suitable target for anti-cancer immunotherapy. It is known that the MAPK pathway control the proliferation of cancer cells, including melanoma cells.[18] The MAPK pathways represent the most frequently mutated signaling pathways in melanoma, and the high prevalence of dysregulation of this pathway has provided a rationale for the development of target-based therapeutics.[27] Cross-talk between FOXM1 and the MAPK pathway has also been demonstrated in malignant melanoma. Therefore, we have conducted the MEK siRNA analysis in four melanoma cell lines, MeWo cells, which are wild-type for both BRAF and NRAS, MM-LH cells, which are wild-type for both BRAF and NRAS, SK-MEL28 cells, which had the BRAFV600E mutation and are wild-type for NRAS, and VM115 cells, which had the BRAFV600E mutation and are wild-type for NRAS, to determine whether MEK1 siRNA affect FOXM1 expression. We found that MEK1 siRNA downregulated the expression of FOXM1, together with p-MEK, in three melanoma cells (MeWo cells, MM-LH cells and VM115 cells). (Fig 6) These data suggest the possibility that FOXM1 is activated by the MAPK pathway in these melanoma cell lines. There were no relationships with BRAF status.

### The AKT activity is not affected by FOXM1 siRNA in melanoma cells

Recently, the combined use of BRAF and MEK inhibition has become a new standard for inhibiting the MAPK pathway in patients with advanced BRAF mutant melanoma. However, the problem of acquired resistance has become a major stumbling block to obtaining long-term disease control. [28] We therefore considered the blockade of an alternate pathway to be important. Furthermore, Wang et al. reported that the downregulation of AKT by siRNA inhibited FOXM1 expression and that the overexpression of AKT increased FOXM1 expression in prostate cancer. [15] For the above-stated reasons, we examined whether FOXM1 expression was regulated by AKT in melanoma cells.

**Fig 7:**
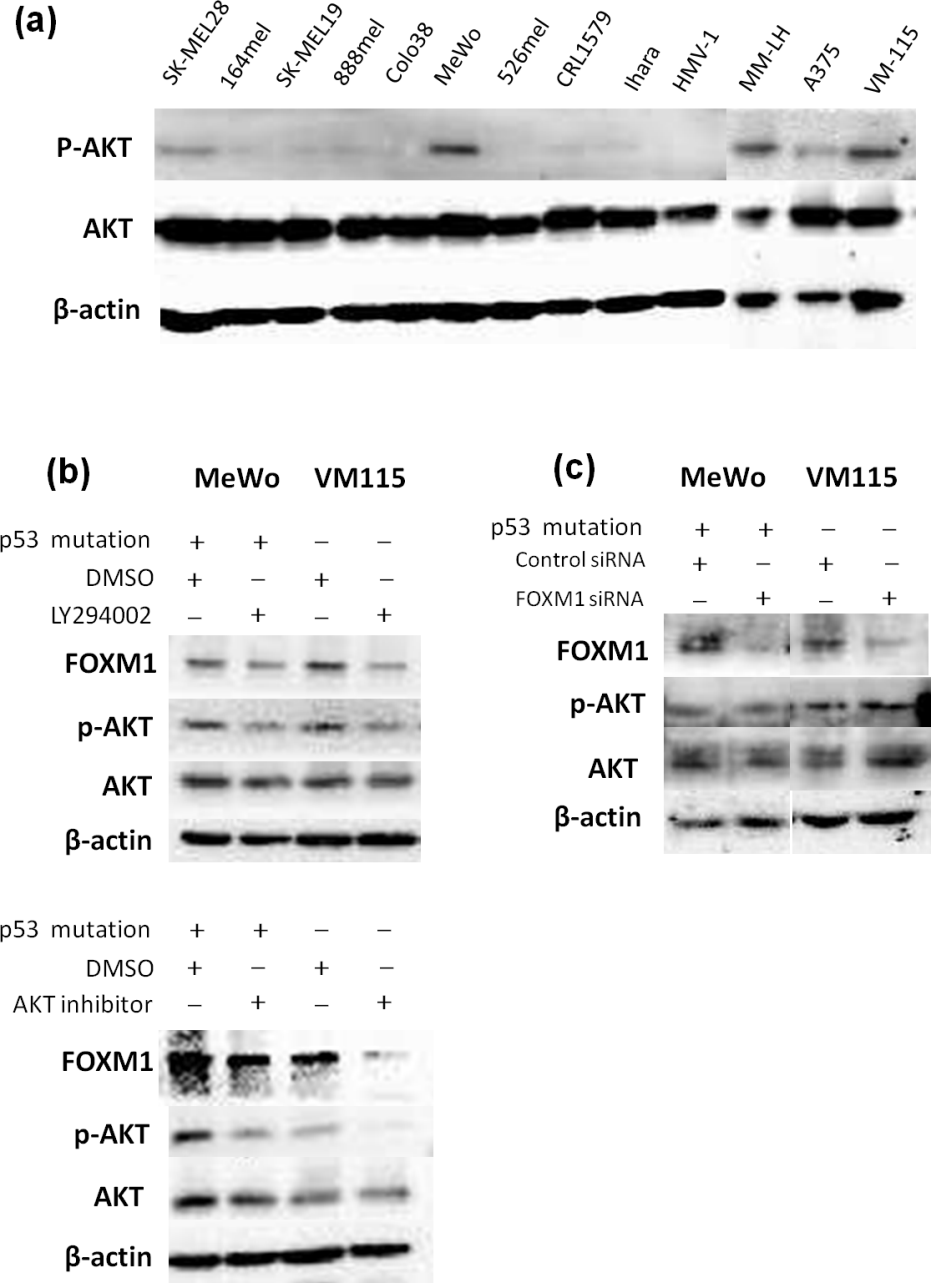
PI3K/AKT signaling pathway blockade in melanoma cells using the PI3K inhibitor, LY294002, an AKT inhibitor and FOXM1 siRNA. (a) The expression of activated AKT was assessed by a Western blotting analysis using phospho-specific anti-AKT antibodies. (b) The results of the Western blotting analysis of the cell lysates from the human melanoma cell lines treated with LY294002 (30 μM) and an AKT inhibitor (20 μM) for 24 hours. The levels of FOXM1, p-AKT (ser 473) and AKT were determined. (c) Melanoma cell lines were transfected with control or FOXM1 siRNA, and a Western blotting was carried out with p-AKT (ser 473), AKT and FOXM1 antibodies 72 hours after treatment.

First, to determine whether AKT was activated in melanoma cell lines, the expression of activated AKT was assessed using a Western blotting analysis with a phospho-specific anti-AKT antibody. In some melanoma cell lines, AKT was highly phosphorylated compared with that observed in the other cell lines (Fig 7A). We thus conducted LY294002 (a PI3K inhibitor) and AKT inhibitor studies in two melanoma cell lines in which AKT was highly phosphorylated: MeWo cells, which have a p53 mutation and VM115 cells, which have functional p53. We found that LY294002 and the AKT inhibitor reduced the expression of FOXM1 (Fig 7B), thus suggesting that FOXM1 is regulated by the PI3K/AKT pathway in melanoma cells. However, the downregulation of FOXM1 by siRNA did not affect the expression levels of p-AKT in the melanoma cells (Fig 7C).

## Discussion

FOXM1 has been reported to be overexpressed in various cancers and may be a suitable target for immunotherapy [9]. To our knowledge, this is the first study to demonstrate the FOXM1 expression in melanocytic lesions. In this study, we found that the metastatic melanoma samples exhibited significantly higher mRNA expression levels of FOXM1 (*p* = 0.004). In the immunohistochemical analyses, FOXM1 was overexpressed in 49% of the primary melanomas and 67% of the metastatic melanomas, whereas a markedly lower rate of expression was observed in the benign melanocytic nevi. The nevus tissues expressed some FOXM1 mRNA, however, there were relatively lower levels of FOXM1 protein expression. It also revealed that the FOXM1 expression levels are not absolutely specific to malignant melanoma, and can also be detected in nevi. Previous studies have shown that HLA class I molecules are downregulated in nevus tissue samples [29], therefore, nevi are isolated from the adaptive immune response. We think the expression of FOXM1 in nevi can be considered accepted. Cancer-Testis antigens, like MAGE and NY-ESO, can be considered good therapeutic targets for immunotherapy and there are many clinical trials using Cancer-Testis antigens. It is known that the cancer-testis antigens are expressed not only in the cancer tissues but also in the testis. However, it does not become a major problem because the testis is isolated from the adaptive immune system. The testis is devoid of HLA-class I molecules and cannot present antigens to the T cells [30]. As 49% of the primary malignant melanomas and 67% of the metastatic melanomas evaluated in this study expressed FOXM1, it would be inappropriate for all patients with melanoma to be treated with FOXM1-targeted immunotherapy. In fact, we demonstrated that immunotherapy employing multiple tumor-associated antigens is more effective than that employing single tumor-associated antigens [31]. Furthermore, multiple peptides cocktail vaccine (KOC1, FOXM1 and KIF20A) has already been tested for the patients with refractory pediatric sarcoma in phase I study. In future clinical trials, the use of multiple antigen-targeted immunotherapies, including FOXM1, should be also considered in melanoma. We believe that the combination of immunocheckpoint blockades and antigen specific immunotherapy, utilizing FOXM1, will therefore make immunocheckpoint blockades more effective. Moreover, it has been shown that higher expression of FOXM1 is associated with a poor prognosis in several cancers [32–34]. In melanoma patients, it has been shown that the five- and 10-year survival rates decrease significantly as the tumor thickness increases [23]. Since we found that there was significant correlation between the FOXM1 expression and tumor thickness (*p* = 0.046). We also found that there was a significant difference in the overall survival between the FOXM1-positive patients and negative patients (*p* = 0.024). It can be assumed that the FOXM1 expression is correlated, directly or indirectly, with the prognosis of melanoma patients.

It is known that the MAPK and PI3K/AKT pathways represent the most frequently mutated signaling pathways in human cancers, including malignant melanoma. It has been reported that up to 70% of melanomas carry the BRAFV600E mutation [19], and 70% have elevated AKT phosphorylation [20]. FOXM1 has been shown to have cross-talk with the MAPK pathway in malignant melanoma [17, 18]. Cross-talk between the AKT pathway and the FOXM1 pathway has been demonstrated in osteosarcoma and prostate cancer [21, 22]. We conducted an MEK siRNA analysis in four melanoma cell lines, and found that MEK1 siRNA downregulated the expression of FOXM1, together with p-MEK, in two melanoma cells. (Fig 6) We showed that the inactivation of AKT by PI3K and AKT inhibitors decreased the expression levels of FOXM1 in melanoma cell lines. Furthermore, we found that the downregulation of FOXM1 by FOXM1-specific siRNA inhibited the proliferation of melanoma cells *in vitro*. In patient samples, we found that 55.2% of the cases that were positive for FOXM1 were also positive for BRAFV600E, and 27.6% of the cases that were positive for FOXM1 were also positive for phospho-AKT, meaning that the expression of FOXM1 was correlated with the BRAF mutation status and/or the status of AKT phosphorylation in the patient samples (Table 2).

These data suggest that FOXM1 may be an ideal target for anti-cancer immunotherapy.

Our results have some limitations because of the small number of samples examined by this study. Therefore, further studies with a larger sample number are needed in order to confirm whether FOXM1 is a new therapeutic target for melanoma.
